# Mass spectrometry quantification of clusterin in the human brain

**DOI:** 10.1186/1750-1326-7-41

**Published:** 2012-08-20

**Authors:** Junjun Chen, Meiyao Wang, Illarion V Turko

**Affiliations:** 1 Institute for Bioscience and Biotechnology Research, University of Maryland, Rockville, Maryland, 20850, USA; 2 Analytical Chemistry Division, National Institute of Standards and Technology, Gaithersburg, Maryland, 20899, USA

**Keywords:** Clusterin, QconCAT, Multiple reaction monitoring, Human brain, Alzheimer’s disease

## Abstract

**Background:**

The multifunctional glycoprotein clusterin has been associated with late-onset Alzheimer’s disease (AD). Further investigation to define the role of clusterin in AD phenotypes would be aided by the development of techniques to quantify level, potential post-translational modifications, and isoforms of clusterin. We have developed a quantitative technique based on multiple reaction monitoring (MRM) mass spectrometry to measure clusterin in human postmortem brain tissues.

**Results:**

A stable isotope-labeled concatenated peptide (QconCAT) bearing selected peptides from clusterin was expressed with an *in vitro* translation system and purified. This clusterin QconCAT was validated for use as an internal standard for clusterin quantification using MRM mass spectrometry. Measurements were performed on the human postmortem frontal and temporal cortex from control and severe AD cases. During brain tissues processing, 1% SDS was used in the homogenization buffer to preserve potential post-translational modifications of clusterin. However, MRM quantifications in the brain did not suggest phosphorylation of Thr^393^, Ser^394^, and Ser^396^ residues reported for clusterin in serum. MRM quantifications in the frontal cortex demonstrated significantly higher (*P* < 0.01) level of clusterin in severe AD group (39.1 ± 9.1 pmol/mg tissue protein) in comparison to control group (25.4 ± 4.4 pmol/mg tissue protein). In the temporal cortex, the clusterin levels were not significantly different, 29.0 ± 7.9 pmol/mg tissue protein and 28.0 ± 8.4 pmol/mg tissue protein in control and severe AD groups, respectively.

**Conclusions:**

The proposed protocol is a universal quantitative technique to assess expression level of clusterin. It is expected that application of this protocol to quantification of various clusterin isoforms and potential post-translational modifications will be helpful in addressing the role of clusterin in AD.

## Background

Genome-wide associated studies have linked the *CLU* gene with the risk of developing late-onset Alzheimer’s disease (AD) [1,2]. *CLU* encodes clusterin, a multifunctional glycoprotein, whose involvement in AD has been discussed since increased clusterin expression in AD hippocampus was first reported two decades ago [3]. Subsequent Western blot and ELISA quantifications pointed to increased levels of clusterin in AD hippocampus and in frontal cortex [4], cerebrospinal fluid [5], and plasma [6]. However, other Western blot studies demonstrated that levels of clusterin did not differ significantly between control and AD cases in the frontal cortex, temporal cortex, or thalamus in postmortem human brain [7,8]. The explanation for these differences is unclear, but might originate from the variety of clusterin antibodies used and availability of clusterin epitopes in the different biological samples examined. The challenge of understanding the role of clusterin in AD goes beyond the quantification of protein levels. Clusterin has alternative splicing variants, single nucleotide polymorphisms, and post-translational modifications [9,10], which are difficult to ascertain with immunological methods and likely associated with development of AD. Overall, the availability of a reliable quantitative approach is needed to facilitate clarification of the role of clusterin in AD.

The application of multiple reaction monitoring (MRM) mass spectrometry to proteomics has greatly enhanced the selectivity and accuracy of protein quantification. Absolute quantification using MRM assay relies on isotope-labeled internal standards, which can be peptides [11], concatenated peptides (QconCAT) [12], or full-length proteins [13]. However, utilization of any of these internal standards has limitations. Isotope-labeled peptides provide the most high-throughput analysis, although have the highest chance of serious quantitative discrepancies between analyte and standard. Isotope-labeled full-length proteins are perfectly quantitative as internal standards, but do not support high-throughput analysis due to poor availability. QconCATs have a strong potential to be perfectly quantitative and support high-throughput analysis at the same time [14,15].

QconCATs are similar to analyte proteins, but not identical. Potentially, this can result in incomplete proteolysis of either the QconCATs or the analyte proteins and therefore affect quantification. There are two main determinants of the rate of proteolysis. First is the composition of amino acid residues around the cleavage site. The second is the tight folding of some proteins that makes them intrinsically resistant to proteolysis. We addressed these issues by (i) incorporating into the QconCATs the original flanking sequences from the proteins that surround the tryptic fragments (Q-peptides) to create an identical amino acid composition around the cleavage site, and (ii) initial treatment of biological samples with a SDS-containing buffer to completely unfold proteins. SDS in the homogenization buffer has the added advantage of preserving post-translational modifications that may be present in target proteins. The final optimized protocol was used to assess the levels of clusterin and levels of potential clusterin phosphorylation in the specimens of human brain from control and severe AD donors. The data obtained expand our understanding of the role(s) clusterin may play in AD.

## Results and discussion

### Flanking regions for Q-peptides

**Figure 1:**
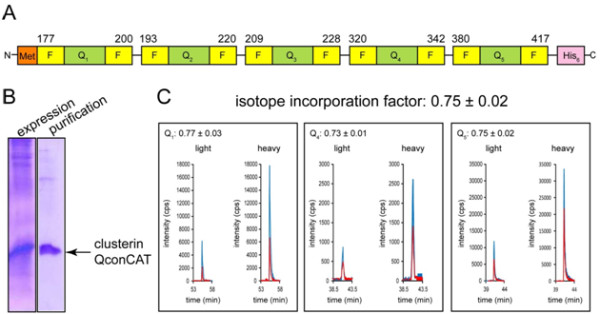
**Design, expression, and characterization of clusterin QconCAT. **(**A**). Clusterin QconCAT includes five Q peptides (shown in *green*) with 6-amino acid long flanking regions (shown in *yellow*) concatenated into the sequence with N-terminal Met and C-terminal His_6_-tag. (**B**) Coomassie R250 stained clusterin QconCAT after 15% polyacrylamide gel separation. (**C**) Stable isotope incorporation into the clusterin. MRM spectra for two representative transitions per Q_1_, Q_4_, and Q_5_ peptides are shown. The pair transitions for light (unlabeled) and heavy (labeled) form of each Q peptide are color coordinated. Isotope incorporation factor for clusterin QconCAT was calculated based on combined data for all three peptides and presented as mean ± SD.

Often, the Q-peptides in the QconCAT are directly adjacent to each other. Thus, the flanking regions of Q-peptides in the standard and analyte are different and proteolysis may occur at a different rate for the QconCAT and analyte. To avoid potential quantitative discrepancy, the incorporation of native flanking sequences into the QconCATs was first proposed by Kito *et al.*[16] and further used by Nanavati *et al.*[14]. At the same time, evaluation of QconCATs without flanking sequences demonstrated that the digestion protocol had a significant impact on final quantification [17]. Figure 1A illustrates the design of our QconCAT for clusterin with five Q-peptides carrying 6-amino acid long extensions from their natural sequences on both sides of the Q-peptides. This QconCAT was expressed as an isotope-labeled protein and purified using a C-terminal His_6_-tag (Figure 1B). The His_6_-tag is separated from the last Q-peptide by the flanking sequence specific for that Q-peptide and therefore will not affect the rate of proteolytic release. Additionally, the presence of flanking sequences eliminates concerns about dibasic cleavage sites and acidic residues at P2’ position and allows for greater flexibility in QconCAT design.

Optimal MRM transitions for Q-peptides were experimentally selected from analysis of clusterin QconCAT tryptic digestion. These transitions were then used to determine the level of stable isotope incorporation in the clusterin QconCAT. Signals from Q_2_- and Q_3_-peptides were low and these two peptides were not used for further quantifications. Figure 1C shows representative extracted ion chromatograms for Q_1_, Q_4_, and Q_5_ peptides. It is evident that both light and heavy versions of the peptides are present. This is presumably due to contaminating unlabeled Arg or Lys amino acids, or synthesis, in the *in vitro* kit. The isotope incorporation factor, α, is calculated as the ratio of the area of the labeled peak to the sum of the unlabeled and labeled peaks. The two strongest transitions per peptide were used to calculate isotope incorporation for each peptide. Combined data for these three peptides resulted in a final value of 0.75 ± 0.02. A single point calibration was used to assign a concentration value based on the following formula:

(1) $$\text{clusterin}\;\left(\text{pmol/mg}\right)=\left[\left(\frac{\text{A}}{\text{S}}+1\right)\text{x}\;\alpha -1\right]\;\text{x}\frac{\text{standard}\;\left(\text{pmol}\right)}{\text{tissue protein}\;\left(\text{mg}\right)}$$

where A is the analyte peak area, S is the standard peak area, and α is the isotope incorporation factor. The equation functions to shift the light component of the Q peptide from the analyte peak area to the standard peak area proportionally based on the isotope incorporation factor.

The MRM transitions used for all following quantifications are summarized in Table 1.

**Table 1:** Peptides and MRM transitions used for the quantification of clusterin

	**MRM transitions (*m/z*)**			
**Peptides**	**Precursor**		**Product**	
ASSIIDELFQDR (Q_1_ ,light)	697.35 (+2)	678.40 (y^5^,+1)	922.43 (y^7^,+1)	1035.51 (y^8^,+1)
ASSIIDELFQD**R** (Q_1_ ,heavy)	702.36 (+2)	688.40 (y^5^,+1)	932.43 (y^7^,+1)	1045.52 (y^8^,+1)
ELDESLQVAER (Q_4_ ,light)	644.82 (+2)	602.30 (y^5^,+1)	802.44 (y^7^,+1)	1046.51 (y^9^,+1)
ELDESLQVAE**R** (Q_4_ ,heavy)	649.83 (+2)	612.30 (y^5^,+1)	812.45 (y^7^,+1)	1056.52 (y^9^,+1)
VTTVASHTSDSDVPSGVTEVVVK (Q_5_ ,light)	772.06 (+3)	507.80 (y^10^,+2)	773.48 (y^7^,+1)	1014.58 (y^10^,+1)
VTTVASHTSDSDVPSGVTEVVV**K** (Q_5_ ,heavy)	774.74 (+3)	511.80 (y^10^,+2)	781.49 (y^7^,+1)	1022.60 (y^10^,+1)

[^13^C_6_,^15^ N_2_]-Lys and [^13^C_6_,^15^ N_4_]-Arg in labeled peptides are shown in *bold*. For MRM transitions, c*harge* for precursor ions*,* and, and the *type* and *charge* for product ions are shown in parentheses.

### SDS-containing buffer for sample processing

QconCATs are artificial proteins, which are not expected to be folded into higher order structures. Nevertheless, on the level of QconCAT design, we selected only those peptides from clusterin which are not involved in *β*-sheet formation in the native clusterin to avoid a potential resistance to proteolysis. To ensure full unfolding of the native clusterin and equal proteolytic rates for analyte (native clusterin) and internal standard (clusterin QconCAT), 25 mM NH_4_HCO_3_ containing 1% (m/V) SDS was selected as a homogenization buffer for tissue processing. In addition, SDS-containing buffer for biological sample processing immediately terminates any enzymatic reactions that can change the levels of present post-translational modifications. After supplementation with QconCAT and alkylation of Cys residues, samples were precipitated with chloroform/methanol to remove SDS, biological lipids, and by-products of alkylation. The resulting pellet of pure and fully unfolded proteins was further proteolysed and analyzed by MRM.

### Validation of proteolytic digestion

To determine the completeness of digestion, we performed trypsin digestions of human recombinant clusterin (purity > 95%, ProSpec-Tany Technogene, Ness Ziona, Israel) and clusterin QconCAT mixed in different ratios. Table 2 demonstrates that, based on three peptides and two different ratios, the average difference between mixed concentrations and actually measured is approximately 16%. In addition to the rate of proteolysis, this combined error also includes the errors generated by mixing of clusterins and the MRM assay itself. Overall, this difference is very acceptable for quantification and the data confirm that proteolytic digestion of our clusterin QconCAT accurately reproduces proteolytic digestion of native full-length clusterin.

**Table 2:** Validation of proteolytic digestion

	**Recombinant clusterin/QconCAT clusterin (pmol/pmol)**	
	**Mixed**	**Measured**
ASSIIDELFQDR (Q_1_)	1.0	1.2 ± 0.1
	2.0	2.4 ± 0.2
ELDESLQVAER (Q_4_)	1.0	1.3 ± 0.2
	2.0	1.9 ± 0.3
VTTVASHTSDSDVPSGVTEVVVK (Q_5_)	1.0	1.2 ± 0.2
	2.0	2.4 ± 0.3
consensus	1.50 (100%)	1.73 (116%)

The measurements were performed for three experimental replicates by monitoring three transitions per individual peptide and presented as mean ± SD. For consensus, the mean measured ratio is presented as a percentile of mean mixed ratio.

### Quantification of clusterin

**Figure 2:**
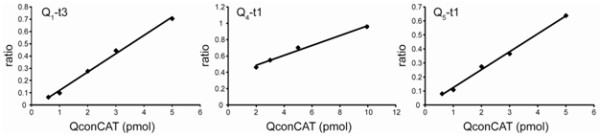
**Representative standard curves for Q**_**1**_**, Q**_**4**_**, and Q**_**5 **_**peptides.** The area ratio of heavy to light peaks for a selected transition was plotted versus suplemented clusterin QconCAT amount for each Q peptide. Transitions: Q_1_-t3 is 697.35/1035.51 and 702.36/1045.52; Q_4_-t1 is 644.82/602.30 and 649.83/612.30; Q_5_-t1 is 772.06/507.80 and 774.74/511.80.

The methodological improvements described above demonstrated that flanking sequences for Q-peptides in QconCAT in combination with full-unfolding of native proteins in 1% SDS prevent potential inconsistency in the tryptic digestion and reduce the bias in quantification. To finalize the protocol for clusterin quantification in brain tissues, the dynamic range and limit of quantification (LOQ) were determined in temporal cortex homogenates. The standard curves were generated for Q_1_-, Q_4_-, and Q_5_-peptides by mixing different concentrations of labeled clusterin QconCAT (0.1 – 10 pmoles range) into the 0.2 mg of temporal cortex homogenate. The data were plotted for every individual transition and showed linearity and low scatter. Figure 2 shows a representative standard curve for each Q peptide. LOQ was defined as the lowest standard point of the curve that could be measured with a coefficient of variance less than 20%. The LOQ was 3 pmol/mg of tissue protein for Q_1_- and Q_5_-peptides. The measurements based on Q_4_-peptide were less sensitive with LOQ equal to 10 pmol/mg of tissue protein. For this reason, Q_4_-peptide was not used for the following quantifications in the human brain.

Clusterin was quantified (summarized in Table 3) in human frontal and temporal cortex brain samples obtained from two groups of donors, control with no or minimal AD changes (clinical dementia rating 0) and severe AD cases (clinical dementia rating 3) [for demographic information of the donors, see Additional file 1. Notably, the measurements based on the Q_1_-peptide (ASSIIDELFQDR) and those based on Q_5_-peptide (VTTVASHTSDSDVPSGVTEVVVK) are similar. Phosphorylation of Thr^393^, Ser^394^, or Ser^396^ in VTTVASHTSDSDVPSGVTEVVVK has been reported for clusterin present in serum [10]. Our protocol for sample processing includes tissue homogenization in the presence of 1% SDS which should prevent loss of post-translational modifications. If any modifications were present in Q_5_, the measurements based on Q_5_ would be lower (than based on Q_1_) proportionally to the level of modification. However, we did not observe a statistically significant difference between quantifications based on Q_1_ and Q_5_. This may point to a very low level of Thr^393^, Ser^394^, or Ser^396^ phosphorylation in the brain, if any.

**Table 3:** Quantification of clusterin in the human brain tissues

	**Clusterin, pmol/mg tissue protein**			
	**Frontal cortex**		**Temporal cortex**	
	**Control**	**Severe AD**	**Control**	**Severe AD**
ASSIIDELFQDR (Q_1_)	26.0 ± 4.2	39.6 ± 9.0	29.5 ± 7.4	24.2 ± 8.5
VTTVASHTSDSDVPSGVTEVVVK (Q_5_)	24.8 ± 4.7	38.6 ± 9.3	27.8 ± 7.9	29.3 ± 7.7
consensus	25.4 ± 4.4	39.1 ± 9.1	29.0 ± 7.9	28.0 ± 8.4

Each control and severe AD group includes six human donors. The concentration was calculated for three experimental replicates by monitoring three transitions per individual peptide and presented as mean ± SD. The monitored transitions are summarized in Table 1. The reported phosphorylated Thr and Ser residues [10] are underlined. For the consensus, the data for both peptides were combined and presented as mean ± SD. Clusterin concentrations in frontal cortex were significantly different (*P* < 0.01) using a *t* test comparison for the control and severe AD groups, whereas clusterin concentrations in temporal cortex were not significantly different.

The second important observation is that the level of clusterin in frontal cortex is significantly higher (*P* < 0.01) in severe AD (39.1 ± 9.1 pmol/mg tissue protein) in comparison to control (25.4 ± 4.4 pmol/mg tissue protein) while the clusterin levels in the temporal cortex are not significantly different, 29.0 ± 7.9 pmol/mg tissue protein and 28.0 ± 8.4 pmol/mg tissue protein in control and severe AD groups, respectively. These quantifications point to a selective increase of clusterin level in the specific region of AD brain.

## Conclusions

We have developed an antibody-free protocol for clusterin quantification in tissue samples. The protocol includes tissue homogenization in 1% SDS that will be helpful in discovery and validation of clusterin isoforms and post-translational modifications. The subsequent chloroform/methanol precipitation is a simple approach to remove SDS before trypsinolysis and MRM. In addition, we have used isotope-labeled QconCAT with original flanking regions for every Q-peptide and demonstrated that this internal standard entirely matched to full-length clusterin in the MRM quantitative assay. Subsequent measurements of clusterin in frontal and temporal cortex from control and severe AD donors showed that the proposed protocol can be used to address the role of clusterin in AD.

## Methods

### Materials

Expressway cell-free *E.coli* expression system was from Invitrogen (Carlsbad, CA). L-[^13^C_6_,^15^ N_2_]lysine and L-[^13^C_6_,^15^ N_4_]arginine (>95% purity) were from Spectral Stable Isotopes (Columbia, MD). The *DC* Protein Assay kit was from Bio-Rad Laboratories (Hercules, CA). Sequencing grade modified trypsin was obtained from Promega Corp. (Madison, WI). Recombinant human clusterin (purity > 95%) was from ProSpec-Tany Technogene, Ness Ziona, Israel. All other chemicals were purchased from Sigma-Aldrich (St. Louis, MO).

### Design, expression and purification of clusterin QconCAT

A synthetic gene encoding 131 amino acids composed of the sequence MQDHFSRASSIIDELFQDRFFTREPDRFFTREPQDTYHYLPFSLPHRRPHFFFFSLPHRRPHFFFPKSRIVRSQAKLRRELDESLQVAERLTRKYNDQYYLRVTTVASHTSDSDVPSGVTEVVVKLFDSDP was synthesized by Integrated DNA Technologies (Coralville, Iowa). The underlined segments represent the signature proteotypic peptides of clusterin (Q-peptides). The synthetic clusterin gene was cloned into the pEXP5CT/TOPO expression vector in-frame to the C-terminal His_6_-tag. Stable isotope-labeled clusterin QconCAT was expressed using an *in vitro* translation kit (Invitrogen) according to the manufacture's protocol. L-[^13^C_6_,^15^ N_2_]lysine and L-[^13^C_6_,^15^ N_4_]arginine were added to the amino acid mixture to replace unlabeled lysine and arginine. After *in vitro* translation, the labeled clusterin QconCAT was purified by nickel-nitrilotriacetic acid resin in batch mode (Qiagen, Valencia, CA). Finally the purified QconCAT was loaded onto the SpinTrap G-25 spin column (GE Healthcare, Waukesha, WI) to exchange buffer into 25 mM NH_4_HCO_3_ with 1% (m/V) SDS. The protein concentration of clusterin QconCAT was measured in the presence of 1% (m/V) SDS using the *DC* protein Assay kit and bovine serum albumin as a standard. The final clusterin QconCAT was aliquoted and kept frozen at −80°C.

### Human tissues

Samples of frontal cortex were received from the Washington University School of Medicine Alzheimer’s Disease Research Center. Samples of temporal cortex were obtained from the Alzheimer's Disease Center, Boston University. All brain specimens were collected from de-identified donors following informed consent of the respective families.

### Processing of samples

The minced brain tissue was placed in 25 mM NH_4_HCO_3_/1% (m/V) SDS and homogenized by sonication at 30 W using five 10 s continuous cycles (Sonicator 3000, Misonix Inc., Farmingdale, NY). The homogenate was centrifuged at 2000 g for 5 min to remove tissue debris. The supernatant was used to measure total protein concentration in the presence of 1% SDS using the *DC* protein Assay kit and bovine serum albumin as a standard. The supernatant was then aliquoted 0.2 mg of total tissue protein *per* tube and kept frozen at −80°C. During the following experiments, samples of 0.2 mg of total tissue protein were supplemented with 20 mM DTT and various amounts of labeled clusterin QconCAT, ranging from 0.1 pmol to 10 pmol per sample. The mixture was incubated at room temperature for 60 min to allow reduction of cysteines and was then treated with 50 mM iodoacetamide for another 60 min. Alkylated samples were precipitated with chloroform/methanol [18]. The protein pellets obtained were sonicated in 100 μL of 25 mM NH_4_HCO_3_ and treated with trypsin for 15 h at 37°C. The substrate/trypsin ratio was 50:1 (m/m). After trypsinolysis, the samples were dried using a vacuum centrifuge (Vacufuge, Eppendorf AG, Hamburg, Germany).

### LC-MS/MS analysis

Instrumental analyses were performed on a hybrid triple quadrupole/linear ion trap mass spectrometer (4000 QTRAP, ABI/MDS-Sciex) coupled to an Eksigent nanoLC-2D system (Dublin, CA). Separation of peptides was performed with an Eksigent cHiPLC- nanoflex system equipped with a nano cHiPLC column, 15 cm x 75 μm, packed with ReproSil-Pur C18-AQ, 3 μm (Dr. Maisch, Germany). Peptides were eluted over a 29 min-gradient from 15% to 35% acetonitrile, containing 0.1% formic acid at a flow rate of 300 nL/min. The column effluent was continuously directed into the nanospray source of the mass spectrometer. All acquisition methods used the following parameters: an ion spray voltage of 2200 V, curtain gas of 105 kPa (15 psi), source gas of 140 kPa (20 psi), interface heating temperature of 170°C, declustering potential of 76 V for +2 precursor ions and 65 V for +3 precursor ions, collision energy of 30 V for +2 precursor ions and 22 V for +3 precursor ions, and collision cell exit potential of 16 V for +2 precursor ions and 13 V for +3 precursor ions. The dwell time for all transitions was 40 ms.

### Quantitative analysis and validation

The initial list of MRM transitions was selected as described [19] and was experimentally screened for the three most intensive transitions per peptide. These transitions were further used for quantification. The relative ratios of the three transitions monitored in the 25 mM NH_4_HCO_3_ for labeled clusterin QconCAT were similar to those observed by spiking labeled clusterin QconCAT into the biological samples. This indicated no significant interference for the quantification based on selected transitions. The identities of the measured peptides were confirmed based on the retention time of the three MRM peaks from a given peptide and the ratio among the three MRM peaks. The reported value for each group was the mean and SD of each biological replicate using all peptide transitions.

## Competing interests

The authors declare no competing financial interest.

## Authors’ contributions

JC, MW, and IVT conceived and designed the experiments. JC expressed and characterized the isotope labeled clusterin QconCAT. IVT performed MS sample preparation. JC and MW performed MS data acquisition and analysis. IVT wrote the paper. All authors revised the manuscript and gave final approval of the version to be published.

## Supplementary Material

Additional file 1 Donors of temporal and frontal cortex. Click here for file
