# Supplementary material for: Mass spectrometry quantification of clusterin in the human brain
Source: Mol Neurodegener. 2012 Aug 20;7:41. doi: 10.1186/1750-1326-7-41 (PMC3470951; doi:10.1186/1750-1326-7-41)
Supplement: Additional file 1 — Donors of temporal and frontal cortex. [file 1750-1326-7-41-S1.docx]

**Additional file 1. Donors of temporal and frontal cortex**

| Donor ID | Age (y) | Gender | Tissue | Clinical Dementia Rating |
| --- | --- | --- | --- | --- |
| 9499 | 81 | M | temporal cortex | 0 |
| 9517 | 83 | M | temporal cortex | 0 |
| 10387 | 86 | M | temporal cortex | 0 |
| 1103282 | 84 | M | temporal cortex | 0 |
| 1103283 | 84 | M | temporal cortex | 0 |
| 1103284 | 83 | M | temporal cortex | 0 |
| 6041 | 81 | M | temporal cortex | 3 |
| 6053 | 87 | M | temporal cortex | 3 |
| 6058 | 68 | M | temporal cortex | 3 |
| 6060 | 76 | M | temporal cortex | 3 |
| 10442 | 86 | M | temporal cortex | 3 |
| 10542 | 80 | M | temporal cortex | 3 |
| 1 | 90 | F | frontal cortex | 0 |
| 5 | 87 | M | frontal cortex | 0 |
| 12 | 81 | F | frontal cortex | 0 |
| 13 | 87 | M | frontal cortex | 0 |
| 14 | 87 | M | frontal cortex | 0 |
| 18 | 92 | F | frontal cortex | 0 |
| 2 | 79 | M | frontal cortex | 3 |
| 3 | 74 | F | frontal cortex | 3 |
| 8 | 81 | F | frontal cortex | 3 |
| 10 | 91 | F | frontal cortex | 3 |
| 11 | 72 | M | frontal cortex | 3 |
| 19 | 84 | F | frontal cortex | 3 |
